# Plant-like substitutions in the large-subunit carboxy terminus of *Chlamydomonas *Rubisco increase CO_2_/O_2 _Specificity

**DOI:** 10.1186/1471-2229-8-85

**Published:** 2008-07-30

**Authors:** Sriram Satagopan, Robert J Spreitzer

**Affiliations:** 1 Department of Microbiology, Ohio State University, Columbus, OH 43210, USA; 2 Department of Biochemistry, University of Nebraska, Lincoln, NE 68588-0664, USA; 3 DuPont Knowledge Center, ICICI Knowledge Park, Hyderabad 500078, India

## Abstract

**Background:**

Ribulose-1,5-bisphosphate is the rate-limiting enzyme in photosynthesis. The catalytic large subunit of the green-algal enzyme from *Chlamydomonas reinhardtii *is ~90% identical to the flowering-plant sequences, although they confer diverse kinetic properties. To identify the regions that may account for species variation in kinetic properties, directed mutagenesis and chloroplast transformation were used to create four amino-acid substitutions in the carboxy terminus of the *Chlamydomonas *large subunit to mimic the sequence of higher-specificity plant enzymes.

**Results:**

The quadruple-mutant enzyme has a 10% increase in CO_2_/O_2 _specificity and a lower carboxylation catalytic efficiency. The mutations do not seem to influence the protein expression, structural stability or the function in vivo.

**Conclusion:**

Owing to the decreased carboxylation catalytic efficiency, the quadruple-mutant is not a "better" enzyme. Nonetheless, because of its positive influence on specificity, the carboxy terminus, relatively far from the active site, may serve as a target for enzyme improvement via combinatorial approaches.

## Background

Ribulose-1,5-bisphosphate carboxylase/oxygenase (Rubisco) (EC 4.1.1.39) is limited by a low carboxylation rate and competing oxygenase activity that initiates a wasteful photorespiratory pathway leading to the loss of fixed carbon [1]. The CO_2_/O_2 _specificity (Ω) of Rubisco is equal to the ratio of catalytic efficiencies (*k*_cat_/*K*_m_) for carboxylation (*V*_c_/*K*_c_) to oxygenation *V*_o_/*K*_o _[2]. Net photosynthetic CO_2 _fixation would be increased if Rubisco could work faster with greater specificity for CO_2 _[1,2]. Because there is natural variation in Rubisco kinetic constants [3], it may be possible to develop genetic engineering strategies aimed at improving the enzyme by focusing on regions responsible for this variation.

The green alga *Chlamydomonas reinhardtii *is an excellent model for studying plant-like Rubisco enzymes comprised of eight large subunits (~55 kDa, coded by the chloroplast *rbcL *gene) and eight small subunits (~15 kDa, coded by a family of nuclear *rbcS *genes) [1]. Mutants that lack Rubisco function can be maintained with acetate, and both the nuclear *rbcS *and chloroplast *rbcL *genes are amenable to transformation [4,5]. Because *Chlamydomonas *Rubisco has a faster carboxylation rate and lower Ω value than Rubisco enzymes of flowering plants [3], but shares ~90% sequence identity with flowering-plant enzymes, a phylogenetic approach has been initiated to define the structural basis for differences in catalysis [6,7]. There are only 34 residues in the *Chlamydomonas *large subunit that differ from those of 500 flowering-plant sequences [6]. Changing five of these "phylogenetic" residues to those common to flowering plants, and introducing a spinach small-subunit loop, produced a *Chlamydomonas *Rubisco enzyme with catalytic properties more like those of flowering plants than *Chlamydomonas*, including a 12–17% increase in Ω [7]. However, because the Ω value was not identical to that of flowering-plant Rubisco, there must be other residues that also contribute to the differences in Rubisco catalytic properties.

**Figure 1:**
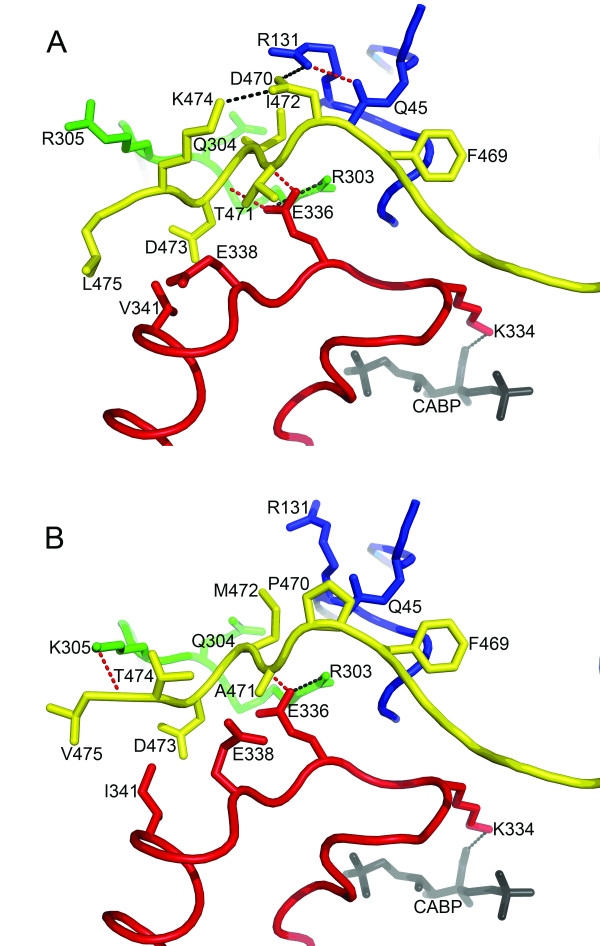
**Comparison of structural interactions at the carboxy-terminal/loop-6 interface in the large subunit of Rubisco from (A) *Chlamydomonas *(1GK8) **[9]** and (B) spinach (8RUC) **[10]. Residues within 4 Å of the divergent carboxy-terminal residues 470, 471, 472, and 474 are shown as sticks. The carboxy terminus (yellow), loop 6 (red), and part of a loop in the amino-terminal domain of a neighboring large subunit (blue) are drawn as ribbons. Residues not in these three structural regions are colored green. The location of the active site is denoted by Lys-334 and the transition-state analog CABP. Potential hydrogen and ionic bonds are indicated by red and black dotted lines, respectively, connecting the participating atoms.

The loop between β-strand 6 and α-helix 6 of the large-subunit α/β barrel folds over the transition-state analog carboxyarabinitol 1,5-bisphosphate (CABP) [8] (Fig. 1). In this closed conformation, the large-subunit carboxy terminus (residues Trp-462 to Leu-475) packs over loop 6 (Fig. 1) [9,10]. In *Chlamydomonas *Rubisco, substitution of conserved Asp-473, which was proposed to be an essential latch residue [11], with either Ala or Glu did not eliminate catalysis but caused a 14–17% decrease in Ω [12]. Deletion of 10 residues from the carboxy terminus of the *Synechococcus *(cyanobacterium) large subunit caused a 38% decrease in Ω, and a group of four plant-like substitutions E470P/T471A/K474T/L475V was reported to cause a 9% increase in Ω [13]. However, this small increase was close to the experimental error of the assays employed [13]. Lengthening the carboxy terminus beyond residue 475 had no effect on Ω [14].

The *Chlamydomonas *carboxy-terminal residues Asp-470, Thr-471, Ile-472, and Lys-474 comprise a set of previously identified "phylogenetic" residues (Fig. 1A) [6] that differ from the set common to flowering plants (Glu-470, Ala-471, Met-472, and Thr-474). The carboxy terminal residues of *Synechococcus*, studied previously [13], comprise a somewhat different set of residues (Thr-471, Lys-474, and Leu-475) relative to those of flowering plants (Ala-471, Thr-474, and Val-475). Therefore, the carboxy terminus of *Chlamydomonas *was changed via directed mutagenesis and chloroplast transformation to that of spinach Rubisco (Fig. 1B) to see whether the catalytic properties common to Rubisco of flowering plants might be obtained.

## Results

### Recovery and phenotype of the mutant strain

The residues equivalent to *Chlamydomonas *Asp-470, Thr-471, Ile-472, and Lys-474 are Glu, Ala, Met, and Thr, respectively, in most flowering-plant sequences [6]. The carboxy terminus of the spinach large subunit is identical in length to that of *Chlamydomonas*, and the availability of the spinach crystal structure [10] allows structural comparison (Fig. 1). However, whereas most plant Rubisco enzymes contain a Glu at position 470, this residue is a Pro in spinach Rubisco. Thus, a *Chlamydomonas *D470P/T471A/I472M/K474T mutant enzyme was created to mimic the large-subunit carboxy terminus of spinach Rubisco.

When the *rbcL*-deletion mutant MX3312 was transformed with the *rbcL*-D470P/T471A/I472M/K474T gene, photosynthesis-competent colonies were recovered. Thus, the substituted residues are not essential for Rubisco expression, assembly, or function in vivo. Furthermore, the mutant strain was indistinguishable from wild type with respect to growth at either 25 or 35°C, eliminating the possibility that the mutant is temperature conditional (data not shown) [15].

### Mutant Rubisco holoenzyme stability

**Figure 2:**
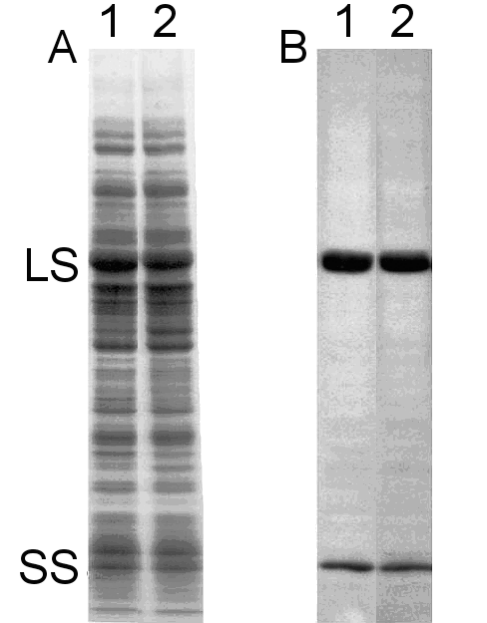
**Protein expression and holoenzyme levels are unaltered in the D470P/T471A/I472M/K474T quadruple mutant**. (A) SDS-polyacrylamide gel electrophoresis and (B) western-blot analysis of total soluble proteins (60 μg/lane) extracted from *Chlamydomonas *wild type (lane 1) and the D470P/T471A/I472M/K474T quadruple mutant (lane 2). The Rubisco large subunit (LS) and small subunit (SS) are indicated.

**Figure 3:**
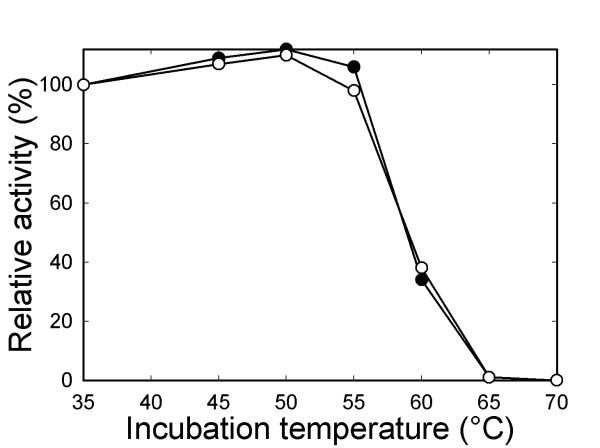
**Carboxy-terminal substitutions do not influence the structural stability of the enzyme in assays performed *in vitro***. Thermal inactivation of Rubisco purified from wild type (○) and the D470P/T471A/I472M/K474T quadruple mutant (●). Rubisco enzymes were incubated at each temperature for 10 min, and then assayed for RuBP carboxylase activity at 25°C. Activities were normalized to the specific activities measured after the 35°C incubation (wild type, 1.7 μmol/min/mg; D470P/T471A/I472M/K474T, 1.2 μmol/min/mg).

To assess the effect of the mutant substitutions on holoenzyme levels in vivo, protein extract was subjected to SDS-PAGE and western-blot analysis. As shown in Fig. 2, the D470P/T471A/I472M/K474T quadruple-mutant strain contains wild-type levels of Rubisco subunits. A wild-type level of holoenzyme could also be purified from the quadruple mutant when cell extract was fractionated on a sucrose-density gradient (data not shown). No difference was detected between the mutant and wild-type enzymes when thermal stability experiments were performed in vitro (Fig. 3). Thus, analysis of the catalytic properties of the quadruple-mutant enzyme would not be expected to be influenced by structural instability during the assays.

### Kinetic properties of the quadruple-mutant enzyme

To determine whether the spinach-like substitutions can confer catalytic properties mimicking those of the spinach enzyme, kinetic constants were determined for the purified mutant enzyme (Table 1). The Ω value of the D470P/T471A/I472M/K474T quadruple-mutant Rubisco enzyme is 10% higher than that of the wild-type *Chlamydomonas *enzyme. Nonetheless, this value is still 17% lower than that of spinach Rubisco (Table 1). Although the Ω value appears to be altered in the correct direction, the other kinetic constants remain quite different from those of spinach Rubisco (Table 1). The increase in Ω is likely the consequence of an increase in *K*_o_/*K*_c _(Table 1). Due to an apparent decrease in *V*_c_/*K*_c_, the D470P/T471A/I472M/K474T quadruple-mutant enzyme would not be a "better" Rubisco [1].

**Table 1:** Kinetic properties of Rubisco purified from *Chlamydomonas *wild type, mutant D470P/T471A/I472M/K474T, and spinach

Kinetic constant	Wild type	Mutant	Spinach
Ω^a ^(*V*_c_*K*_o_/*V*_o_*K*_c_)	61 ± 1	67 ± 2	81 ± 3
*V*_c _^a ^(μmol/h/mg)	119 ± 11	100 ± 7	63 ± 6
*k*_*cat *_^b ^(s^-1^)	2.7 ± 0.2	2.3 ± 0.2	1.4 ± 0.1
*K*_c _^a ^(μM CO_2_)	34 ± 2	40 ± 5	21 ± 1
*K*_o _^a ^(μM O_2_)	417 ± 62	611 ± 119	496 ± 40
*V*_c_/*K*_c _^b^	3.5	2.5	3.0
*K*_o_/*K*_c _^b^	12	15	24
*V*_c_/*V*_o _^b^	5.1	4.5	3.4

^a ^Values are the mean ± (*n*-1) standard deviation of three or more separate enzyme preparations. ^b ^Calculated values.

## Discussion

Substitution of the *Chlamydomonas *carboxy-terminal residues with those of spinach, to produce the D470P/T471A/I472M/K474T quadruple-mutant enzyme, causes a 10% increase in Ω, but the other kinetic constants are not like those of spinach Rubisco (Table 1). Thus, these engineered "phylogenetic" residues [6] do not by themselves account for the differences in catalysis between *Chlamydomonas *and spinach Rubisco. However, there should be other residues in the spinach enzyme that complement for the lower values of K_c _and K_o _(Table 1). Residue 341 (Val in *Chlamydomonas *and Ile in flowering-plant enzymes) is a "phylogenetic" residue in the loop 6, which is in van der Waals contact with the conserved Asp-473 of the carboxy terminus (Fig. 1). Another divergent residue, Arg-305, is also involved in van der Waals contact with the carboxy terminus. The analogous Lys-305 of the spinach enzyme interacts with the carboxy terminus via a single hydrogen-bond (Fig. 1) and a P305K substitution in *Chromatium vinosum *Rubisco causes an 80% increase in carboxylation catalytic efficiency [16]. It is thus likely that the addition of V341I and R305K substitutions to the D470P/T471A/I472M/K474T quadruple mutant would confer kinetic constants that are more like the flowering-plant enzymes.

Engineering of *Synechococcus *Rubisco to produce an E470P/T471A/K474T/L475V mutant enzyme with a spinach-like large-subunit carboxy terminus may also have resulted in a similar, relative increase in Ω [13], but the Ω value of that mutant *Synechococcus *enzyme is still ~10% lower than the Ω value of the *Chlamydomonas *quadruple-mutant enzyme analyzed in the present study. Variation in amino-acid identities at residues 472 and 475 may indicate that these residues are less important than the others. The loss of the E470-R131 and E470-K474 salt bridges in *Synechococcus *Rubisco [17] and D470-R131 and D470-K474 salt bridges in *Chlamydomonas *Rubisco [9] (Fig. 1) may be primarily responsible for the observed increases in Ω [18].

In a recent study, a set of three "phylogenetic" substitutions in the *Chlamydomonas *large subunit (C256F/K258R/I265V), which causes a 10% decrease in Ω [6], was complemented by the addition of two large-subunit phylogenetic substitutions (V221C/V235I) and the loop that resides between β-strands A and B of the spinach small subunit [7,19]. The resultant penta/ABSO enzyme has a ~15% higher Ω value and all other catalytic properties similar to the spinach enzyme [7]. Although the D470P/T471A/I472M/K474T quadruple-mutant enzyme, analyzed in the present study, does not have kinetic constants like those of spinach Rubisco, it does have a 10% increase in Ω (Table 1). If these carboxy-terminal substitutions could increase the Ω value of the penta/ABSO enzyme by 10% without substantially altering other kinetic constants, a *Chlamydomonas *enzyme would be generated with kinetic properties indistinguishable from those of spinach Rubisco [7].

## Conclusion

Such combinatorial approaches may identify only a small number of regions responsible for the differences in kinetic properties between *Chlamydomonas *and spinach Rubisco. These regions may then serve as suitable targets for DNA shuffling and genetic selection aimed at improving Rubisco [20].

## Methods

### Strains and culture conditions

*Chlamydomonas reinhardtii *2137 *mt*^+ ^is the wild-type strain [15]. Mutant MX3312, which has the *rbcL *coding region replaced with the *aadA *gene conferring spectinomycin resistance, was used as the host for chloroplast transformation [12,20]. All strains are maintained in darkness at 25°C on medium containing 10 mM acetate and 1.5% Bacto-agar [15]. For biochemical analysis, cells were grown in 500 ml of liquid acetate medium at 25°C on a rotary shaker at 220 rpm in darkness.

### Directed mutagenesis and transformation

Using the plasmid pUC*rbcL*P as a template [21], the *Chlamydomonas rbcL *gene was PCR-amplified, with the first base of the coding region numbered 1, using forward (bases -169 to -144) and reverse (bases 1404 to 1438) primers. The reverse primer was designed to change codons GAT to CCT, ACT to GCT, ATT to ATG, and AAA to ACA, thereby introducing amino-acid substitutions D470P, T471A, I472M, and K474T, respectively. The reverse primer also contained a 2-base change (TT to AA) following the *rbcL *stop codon that would introduce a *Pac*I recognition site. After PCR amplification, the product was digested with *Bsp*EI and *Pac*I, and cloned into the corresponding sites of a modified plasmid containing the same *Pac*I site (pUC*rbcL*P-*Pac*I). Chloroplast-gene transformation was performed [22,23], and photosynthesis-competent colonies were selected on minimal medium with 80 μmol photons/m^2^/s. Single-colony isolation, followed by PCR and restriction-enzyme analysis, was performed to ensure homoplasmicity of the mutant gene, which was sequenced completely to confirm the mutations.

### Biochemical analysis

Cell extracts were prepared from dark-grown cells and subjected to SDS-PAGE with a 7.5–15% polyacrylamide gradient [24,25]. The proteins in the gels were either stained with Coomassie blue [24] or transferred to nitrocellulose membrane and probed with rabbit anti-*Chlamydomonas *Rubisco IgG (0.5 μg/ml) [19]. Rubisco-subunit IgGs were detected with goat anti-rabbit IgG/alkaline phosphatase conjugate via chemiluminescence (Amersham Pharmacia Biotech) [25,19].

Rubisco holoenzyme was purified from cell extracts of *Chlamydomonas *and spinach (*Spinacea oleracea*, described previously [7]) by sucrose-gradient centrifugation in assay buffer (50 mM Bicine, pH 8.0, 10 mM NaHCO_3_, 10 mM MgCl_2_, 1 mM dithiothreitol) [26]. The kinetic constants of purified and activated enzymes were determined by measuring the incorporation of acid-stable ^14^C from NaH^14^CO_3 _[27]. Ω values of the enzymes (10 μg/0.5-ml reaction) were determined by assaying carboxylase and oxygenase activities simultaneously with 22 μM [1-^3^H]RuBP (15.8 Ci/mol) and 2 mM NaH^14^CO_3 _(5 Ci/mol) in 30-min reactions at 25°C [28,29].

Rubisco thermal stability was assayed by incubating purified and activated enzyme (5 μg) at various temperatures for 10 min [30]. The samples were then cooled on ice, incubated at 25°C for 15 min, and used to initiate reactions in 0.5-ml standard assay mixtures containing NaH^14^CO_3 _(2 Ci/mol) and 0.4 mM RuBP [30].

## Authors' contributions

SS contributed to the conceptualization and design of the study, carried out the experiments and data analysis, and drafted the manuscript. RJS conceived of the study, participated in its design and coordination, and made critical revisions to the draft for intellectual content. Both the authors have read and approve this final manuscript.
